# Letter from Accoucheur. No. 4

**Published:** 1846-01-01

**Authors:** 


### Letter from Accoucheur.—No. 4.

For the Buffalo Medical Journal.

At the commencement of my last letter I remarked, that the presentation of the fœtus in parturition may be ascertained, generally, very early in labor. The following cases may serve as illustrations of some of the difficulties which the practitioner may occasionally meet with.

I attended Mrs. G. in labor. The waters had already discharged themselves, and on examination the os uteri was found fully dilated, disclosing without question, a breech-presentation. As the pelvis was capacious, and the pains were effective, the labor was left to nature. After some hours of unlooked for delay, touching, occasionally, to note the progress of labor, I was not a little surprised to find a tongue protruding from what I had considered the distinctive mark of a breech presentation.—

This occurrence gave quite a *new face* to the affair. Nevertheless, in a short time the child was expelled by natural pains.

I had often repeated this anecdote to my familiar, professional friends, and many years afterward, my old and respected neighbor, Dr. H., called me to a very lingering case of *breech* presentation, in a young woman with narrow pelvis, in labor with her first child; and he jocularly said—"no mistake, there is no tongue here." It turned out, on examination, however, to be a *face* presentation—and the delivery of a dead child was effected after some difficulty, with the forceps. It seemed that my old friend, in his too eager search for a tongue, had converted the fœtus into a young Cyclops.

Dr. ——, sent for me after having been in attendance on a young woman, in labor with her first child 36 hours. He told me the waters were early discharged, the os uteri fully dilated, and the pains were regular, forcible, and strong. To my enquiry, "what is the presentation?" he frankly replied, he did not know. With something like a sneer, I expressed my surprise, as he had had much experience, and was a successful accoucheur. After a careful, close, and critical examination, I was constrained to make the "honorable amend," for I could learn no more than that the pelvis was filled with a globular tumor which obviously contained *bones*. Possessing ourselves of a patch of the external covering of the tumor, it proved to be scalp, and with a pocket knife instead of a trochar, we punctured the tumor, and drew off between two and three pounds of water. The tumor collapsed, and the fœtus was expelled by natural pains, presenting the remains of an enormous head, with shrunken and shrivelled members.

It is somewhat extraordinary that, excepting in one other case of hydrocephalus—though I have known it done many times by my professional acquaintances, I have not found it necessary in 40 years, from malformation, distortion of the pelvis, or any other cause, to lessen the head.

*October*, 1845.

